# SARS-CoV-2 in Pregnancy—A Retrospective Analysis of Selected Maternal and Fetal Laboratory Parameters

**DOI:** 10.3390/ijerph192215307

**Published:** 2022-11-19

**Authors:** Maciej Sobkowski, Beata Pięta, Anna Sowińska, Marlena Grabowska, Katarzyna Koch-Brzozowska, Maciej Wilczak, Agnieszka Bień

**Affiliations:** 1 Department of Mother and Child Health, Poznan University of Medical Sciences, Polna Street 33, 60-535 Poznań, Poland; 2 Department of Informatics and Statistics, University of Medical Sciences, Rokietnicka Street 7, 60-806 Poznań, Poland; 3 Gynecological and Obstetric Hospital of the University of Medical Sciences in Poznan, Polna Street 33, 60-535 Poznań, Poland; 4 District Hospital in Miedzychod, Szpitalna Street 10, 64-400 Międzychód, Poland; 5 Chair of Obstetrics Development, Faculty of Health Sciences, Medical University of Lublin, 4/6 Staszica St., 20-081 Lublin, Poland

**Keywords:** COVID-19, pregnancy, delivery, maternal outcomes, neonatal outcomes

## Abstract

Pregnant women and their neonates belong to the group of individuals with elevated risk for COVID-19 infection. Data on the course of the disease and how it affects the pregnancy and neonatal wellbeing remain conflicting. The aim of the study was to evaluate the effect of SARS CoV-2 infection on the mode of delivery, neonatal condition and selected maternal and fetal laboratory parameters. This was a single-center retrospective case–control study. This dataset was generated using electronic medical records collected by medical personnel. Two groups of patients, hospitalized between April, 2020 and February, 2021, were included in the study: study group (304)—pregnant women with SARS-CoV-2 and control group (*N* = 329)—healthy pregnant women or parturients. Mothers with a severe course of COVID-19 had higher activated partial thromboplastin—APTT (*p* = 0.02), C-Reactive Protein—CRP (*p* = 0.00) and procalcitonin (*p* = 0.032) levels as compared to pregnant women with mild or moderate course of the disease. Neonates born to SARS-CoV-2-infected mothers presented with worse condition at 1 and 5 minutes of life (*p* = 0.000 and 0.00, respectively) and lower Arterial Blood Gas—ABG pH scores (*p* = 0.016). Elective cesarean section is the most common mode of delivery for SARS-CoV2-infected mothers. Emergency cesarean sections are performed at earlier gestational age as compared to vaginal delivery and elective cesarean section. Lower Apgar scores were observed in neonates born to SARS-CoV-2-infected mothers who required oxygen therapy and whose procalcitonin levels were elevated. There is a relationship between more severe course of COVID-19 and APTT, as well as CRP and procalcitonin levels.

## 1. Introduction

Coronavirus disease (COVID-19), which is caused by the SARS-CoV-2 virus, constitutes a threat to the collective health of the global population. The modes of transmission may be airborne and vertical [1,2,3]. Fast transmission and severe clinical course of the disease constitute an unquestionable challenge for the healthcare system worldwide. March 2020 has brought the challenge of providing maternity care in new conditions, overshadowed by the threat of COVID-19 and the associated risks for the pregnancy and labor, its effect on maternal and fetal health, and the necessity of reorganizing hospitals to provide such care to the affected patients. The first guidelines and management protocols for pregnant women with SARS-CoV-2 were issued by the Royal College of Obstetricians and Gynecologists on 13 March 2020 [4]. Despite being a physiological state, pregnancy predisposes women to respiratory complications of a viral infection. Various physiological changes which take place in the immune and cardiopulmonary system increase the probability of a more severe course of a disease caused by a respiratory virus [5,6,7,8,9]. In addition, SARS-CoV-2 infection has been reported to be related to a higher rate of adverse obstetric outcomes such as preeclampsia, preterm birth, and stillbirth [5,6,10].

Complications are more common in SARS-CoV-2-infected pregnant women if COVID-19 occurs in the last month before delivery. The vast majority of the complications are observed in women who were not vaccinated against SARS-CoV-2, whereas the number of complications in women vaccinated during pregnancy is the same as those who were not infected [11]. Multicenter studies and analyses are necessary to assess the relationship between specific risk factors during pregnancy and the course of COVID-19. In order to determine the effect of COVID-19 on the early pregnancy, intrauterine fetal growth, and the risk for miscarriage or stillbirth, it is essential to collect extensive data on the pregnant women, divided by trimesters, and the risk factors. It seems prudent to also include the pre-conception period into the analysis [9]. 

The aim of this retrospective study was to evaluate the effect of SARS CoV-2 infection on the mode of delivery and neonatal condition, and to analyze selected laboratory parameters of the mothers and their neonates. 

## 2. Materials and Methods

The study included a total of 633 women, either pregnant or parturients, hospitalized between April 2020 and February 2021 at the Gynecological and Obstetric Hospital of the Medical University in Poznań (Obstetrics and Gynecology Isolation Unit), Greater Poland Voivodeship, Poland. The study was conducted after obtaining the approval of the hospital director (approval number: 39/2021). This was a single-center retrospective case–control study. This dataset was generated using electronic health records collected by medical personnel. The pregnant women presented at the hospital due to symptomatic infection or signs of labor. The RT-PCR test for SARS-CoV-2 was performed in all women admitted to the hospital. The health records of all pregnant women with SARS-CoV-2 hospitalized at the time of the study were analyzed. A positive test result was one of the inclusion criteria for the study group. The participants were divided into two groups: study group (*N* = 304)—pregnant women or parturients with SARS-CoV-2 and controls (*N* = 329)—healthy pregnant women or parturients. The diagnosis of SARS-CoV-2 using the RT-PCR test constituted the exclusion and inclusion criterion for both groups. The following exclusion criteria were used for both, the study group (diagnosed SARS-CoV-2 infection) and controls (negative SARS-CoV-2 test): multiple pregnancy [12], gestational age of <22 weeks, intrauterine fetal demise, lethal defects in the fetus [13], missing data in the electronic health records from the ESKULAP system (including missing laboratory results). Two women died postdelivery due to complications after SARS-CoV-2 infection.

The following criteria of COVID-19 severity were used:

Asymptomatic or pre-symptomatic infection: positive SARS-CoV-2 result of a virologic test (i.e., a nucleic acid amplification test [NAAT] or an antigen test) but no symptoms which are consistent with COVID-19.

Mild illness: presence of any of the various signs and symptoms of COVID-19 (e.g., fever, cough, sore throat, malaise, headache, muscle pain, nausea, vomiting, diarrhea, loss of taste and smell) but no shortness of breath, dyspnea, or abnormal chest imaging.

Moderate illness: evidence of lower respiratory disease during clinical assessment or imaging and oxygen saturation measured by pulse oximetry (SpO_2_) ≥ 94% on room air at sea level.

Severe illness: SpO_2_ < 94% on room air at sea level, a ratio of arterial partial pressure of oxygen to fraction of inspired oxygen (PaO_2_/FiO_2_) < 300 mm Hg, a respiratory rate > 30 breaths/min, or lung infiltrates > 50%.

Critical illness: respiratory failure, septic shock, and/or multiple organ dysfunction [14].

Blood for the tests was drawn before the delivery. Hemoglobin, Erythrocyte, Leukocyte, Neutrophil, Fibrinogen, APTT, CPR, and Procalcitonin levels were measured in all patients. Additionally, D-dimer level was measured in SARS-CoV-2-positive women. 

Neonatal condition was assessed using the Apgar score and the Arterial Blood Gas (ABG) result. The Apgar scoring system provided a standardized assessment for infants after delivery. The Apgar score comprises five components: (1) color, (2) heart rate, (3) reflexes, (4) muscle tone, and (5) respiration, each of which is given a score of 0, 1, or 2 [15].

**Figure 1:**
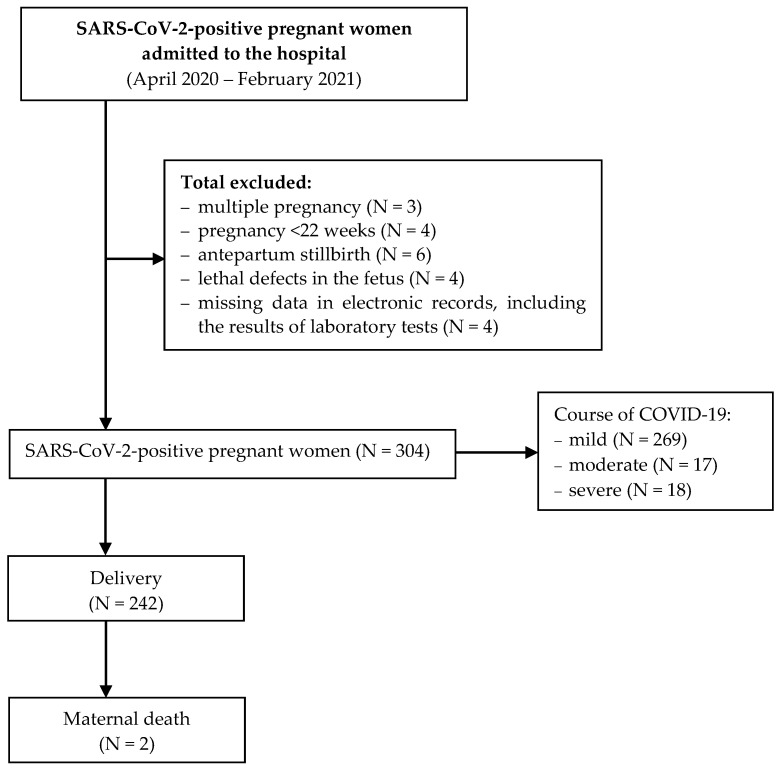
Flow diagram of the exclusions and final analytic samples.

ABG pH test measures acid-base balance in the blood [16]. Upon birth, all neonates were RT-PCR tested for SARS-CoV-2. Detailed selection of the analyzed cases is presented in Figure 1. 

The study material was collected using the hospital IT platform (ESKULAP). ESKULAP is a multi-functional IT system for healthcare entities which is used to collect data about the patient and the process of treatment during hospitalization. The system uses the Oracle database.

### Statistical Analysis

Dell Statistica (data analysis software system), version 13. software.dell.com (Tibco Software Inc. Palo Alto, CA, USA) and Cytel Studio V 11.1.0 (5 January 2016) (Cytel, Waltham, MA, USA)) were used for statistical analysis. Categorical variables were presented as % (*n*). Quantitative variables with normal distribution were presented as mean $\bar{x}$ ± SD, whereas ordinal variables or those with non-normal distribution were presented as median (min-max). The Shapiro–Wilk test was used to assess normality of distribution. Non-parametric tests—Mann–Whitney, Kruskal–Wallis with post hoc Dunn and Spearman’s rank correlation coefficient—were used for ordinal quantitative variables or those with non-normal distribution. Chi-square test with Yate’s continuity correction, chi-square test, Fisher-Freeman-Halton test, and Fisher’s Exact test, with Bonferroni correction for multiple comparisons, were used to analyze the correlations between qualitative variables. The level of statistical significance was set at *p* < 0.05.

## 3. Results

SARS-CoV-2-positive patients were older (*p* = 0.016), at earlier gestational age (*p* < 0.0001), more often presented with hypothyroidism (*p* = 0.019), and cesarean section was more often the mode of delivery in that population (*p* = 0.011) as compared to controls. The highest number of women from the study group had a mild course of COVID-19 (88.49%) and did not require oxygen therapy (89.80%), two women died in the puerperium (0.83%)—Table 1.

**Figure 2:**
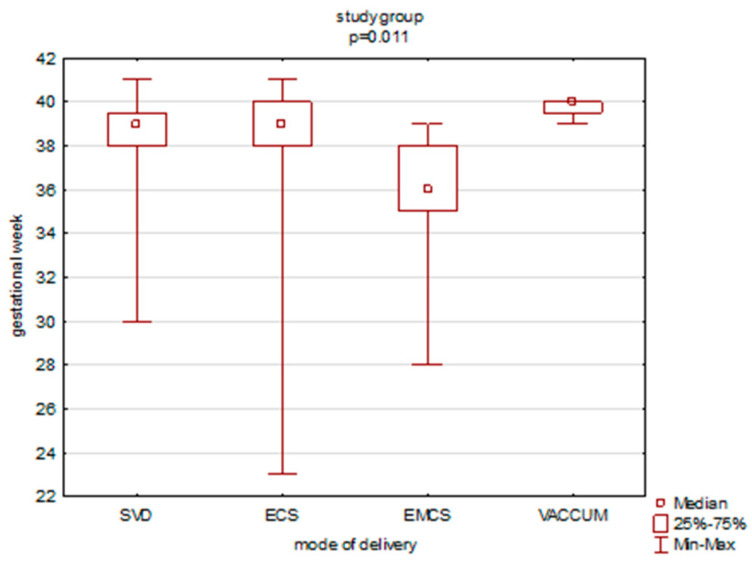
Mode of delivery versus gestational week in the study group. SVD—spontaneous vaginal delivery; ECS—elective cesarean section; EMCS—emergency cesarean section; VACCUM—vacuum extraction.

In the study group, a statistically significant difference between gestational week and mode of delivery was observed. Emergency cesarean section was performed at earlier gestational week as compared to spontaneous vaginal delivery (*p* = 0.03) and elective (*p* = 0.04) cesarean section (Figure 2).

### 3.1. The Effect of SARS-CoV-2 Infection on Selected Maternal Blood Parameters

Statistically significant differences between the study group and controls were found for the following blood parameters: hemoglobin (*p* = 0.00), erythrocyte (*p* = 0.00), leukocyte (*p* = 0.00), neutrophil (*p* = 0.02), fibrinogen (*p* = 0.00), APTT—activated partial thromboplastin time (*p* = 0.00), and CRP—C-Reactive Protein (*p* = 0.00). Hemoglobin level was higher (Me = 7.6) in the study group as compared to controls (Me = 6.8). Erythrocyte level was significantly higher (Me = 4.1) in the study group as compared to controls (Me = 3.6). Higher levels of fibrinogen were found in the study group as compared to controls (median 4.2 vs. 3.7, respectively). Additionally, higher CRP was found in the study group (Me = 7.6) as compared to controls (Me = 4.0). An inverse relationship was observed for leukocyte and neutrophil levels, which were higher in controls (median 12.9 and 75.9, respectively) as compared to the study group (median 9.1 and 74.2, respectively). Median APTT was 28.6 and 30.1 in the study group and controls, respectively (Table 2).

### 3.2. The Effect of COVID-19 Severity on Selected Blood Parameters

**Figure 3:**
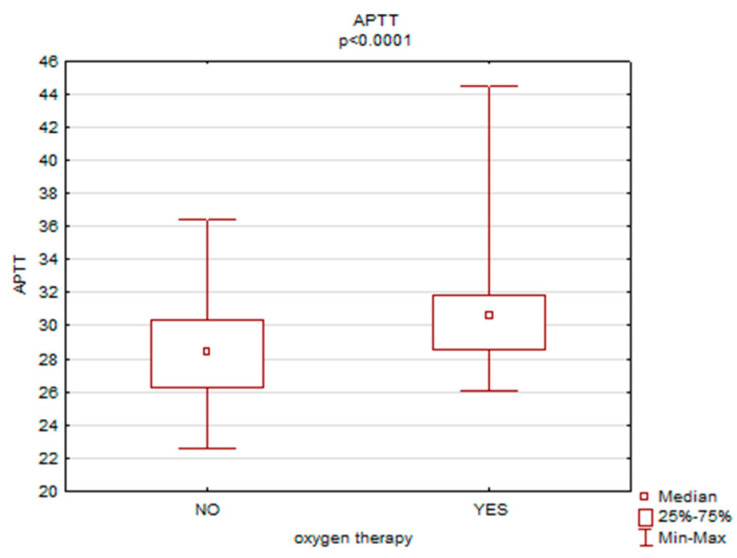
The necessity of oxygen therapy versus APTT levels.

**Figure 4:**
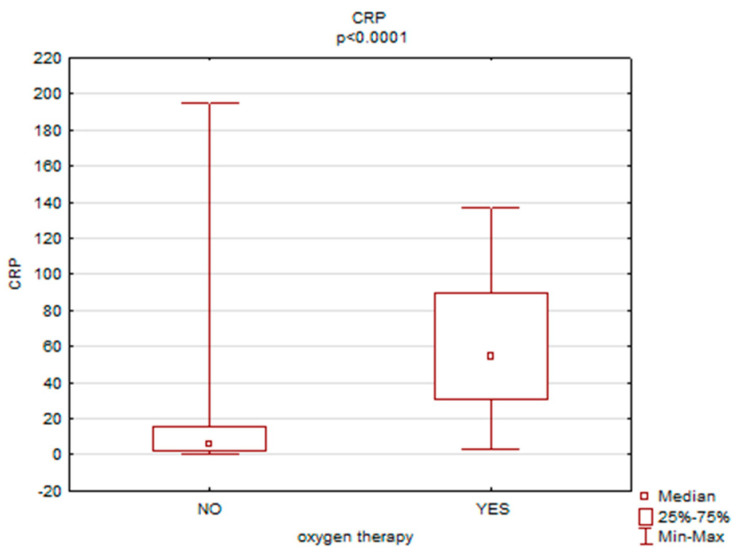
The necessity of oxygen therapy versus CRP levels.

**Figure 5:**
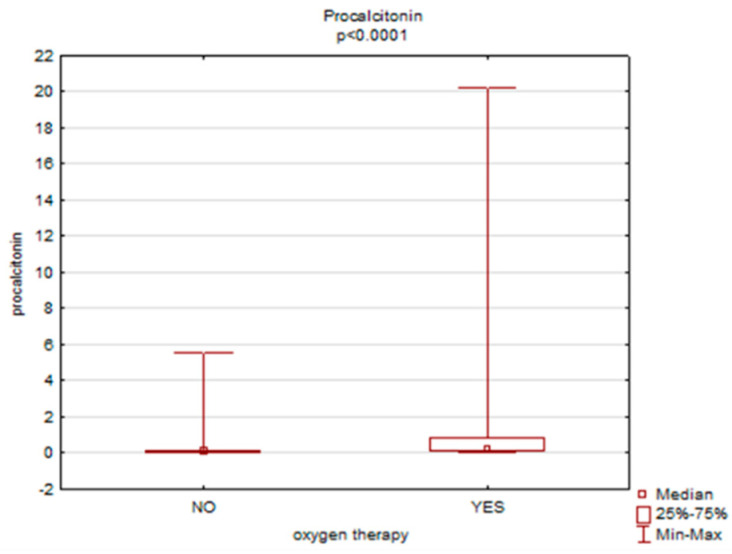
The necessity of oxygen therapy versus procalcitonin level.

A statistically significant relationship was found between the necessity of oxygen therapy and APTT (*p* = 0.00), CRP (*p* = 0.00) and procalcitonin (*p* = 0.00) levels. Women who required oxygen therapy had higher levels of APTT (30.6 vs. 28.4), CRP (54.6 vs. 6.0) and procalcitonin (0.2 vs. 0.04)—Figure 3, Figure 4 and Figure 5.

Pregnant women with severe course of the disease had higher APTT (*p* = 0.02), CRP (*p* = 0.00) and procalcitonin (*p* = 0.032) values as compared to their peers with mild or moderate course of the disease—Table 3.

### 3.3. The Effect of Maternal SARS-CoV-2 Infection on Neonatal Condition at Birth 

Statistically significant differences between both groups in neonatal Apgar scores at 1 and 5 minutes and postdelivery Arterial Blood Gas (ABG) test (pH) were observed. Neonates born to SARS-CoV-2-infected mothers presented with worse condition at 1 and 5 minutes (*p* = 0.000 and 0.00, respectively) of life and lower ABG pH scores (*p* = 0.016) (Table 4). 

Upon birth, all neonates were RT-PCR tested for SARS-CoV-2 and no cases of vertical mother-to-child transmission were found. 

Neonates born to SARS-CoV-2-infected mothers who required oxygen therapy presented with lower Apgar scores at 1 (*p* = 0.00) and 5 (*p* = 0.030) minutes of life.

An inversely proportional correlation was observed between procalcitonin and Apgar score at 1 min (rs = -0.445; *p* = 0.043), i.e., higher concentration of procalcitonin corresponded to lower Apgar score—Table 5.

## 4. Discussion

Perinatology and neonatology have been as affected by the SARS-CoV-2 pandemic as other areas of healthcare. Research into the consequences of coronavirus infection for fetal growth and development, not to mention maternal and neonatal health and wellbeing, continues and a coordinated global effort is necessary to shed more light on the matter. The aim of the study was to retrospectively evaluate the effect of SARS CoV-2 infection on the mode of delivery and neonatal condition and to analyze selected laboratory parameters in the mothers and their neonates.

Data on the correlation between COVID-19 mortality and age have identified age to be the key factor for certain individuals, typically from high- or very high-risk groups and with concomitant diseases. Expectant mothers are members of the high-risk group as pregnancy, despite being a physiological process, is associated with changes in the immune and endocrine systems which increase the risk for viral infections, specifically of the respiratory tract, with a more severe course of the disease [9,17]. In the present study, we found statistically significant differences in age between the two groups: SARS-CoV-2-infected women were older. According to Allotey et al. [9], risk factors for a more severe course of COVID-19 in pregnancy include maternal age, high BMI, and chronic diseases. The prognosis for the severity of the infection is almost 2-fold worse for women with chronic hypertension as compared to their normotensive peers. Gestational diabetes and pre-pregnancy diabetes are also associated with a more severe course of the infection and worse prognosis [9].

We noted that pregnant SARS-CoV-2-infected women were admitted to the hospital at earlier gestational age as compared to non-infected pregnant women. Other authors demonstrated higher incidence of preterm labors in COVID-19-infected patients, irrespectively of disease severity [18,19,20]. In a meta-analysis by Di Mascio et al., preterm labor was observed in 41%, and premature rupture of membranes (PROM) in 19% of the patients [21].

In our study, most patients presented with a mild course of the infection, followed by moderate and severe course. Oxygen therapy was required in 10.2% of the women from the study group. The goal of oxygen therapy is to maintain SpO_2_ at ≥ 95%, while simultaneously decreasing dyspnea and the number of breaths [22]. The patients who required oxygen therapy had higher levels of APTT, CRP and procalcitonin. The values of APTT, CRP and procalcitonin continued to increase as the condition of the patients was gradually deteriorating and were positively correlated with disease severity. Similar observations were reported by Allotey et al. [9] and Boushra et al. [23], who observed an asymptomatic course of COVID-19 in 75%, severe course in 13%, the need of admission to IUC in 4%, and the necessity of mechanical ventilation in 3% of the pregnant women. The women who required admission to ICU and oxygen therapy, or mechanical ventilation, presented with leukopenia and lymphopenia, elevated levels of CRP, D-dimer, lactate dehydrogenase (LDH) and interleukin 6 (IL-6) [9,23]. Higher levels of these parameters are associated with more severe course of the infection, especially the combination of elevated D-dimer and interleukin-6 levels [23,24]. Even though the course of the infection was mild in most women from our study group, with no need of intensive medical care, in some cases the course of the disease may be violent or atypical. 

The decision to perform a cesarean section should always be based on obstetric (fetal or maternal) indications or respiratory capacity, not just on the COVID-19 status. It seems prudent to also take into consideration maternal preferences, as well as safety of the medical personnel, when deciding the mode of delivery [17]. In our study group, elective cesarean section was the most common mode of delivery as compared to spontaneous vaginal delivery, or emergency cesarean section. Emergency cesarean sections were performed at earlier gestational age as compared to vaginal delivery or elective cesarean section. The rate of elective cesarean sections may seem high since data were collected at the beginning of the pandemic. At that time, elective cesarean section was recommended to SARS-CoV-2-infected mothers by the international guidelines [4].

In our study, 5.95% of the women presented with severe infection. Two patients died due to SARS-CoV-2-related complications after childbirth. Lokken et al. [25], who analyzed the severity of COVID-19 in pregnant women in Washington State, demonstrated that expectant mothers are in the high-risk group for SARS-CoV-2 incidence and mortality. The hospital admission rate due to COVID-19 was 3.5-fold higher in the study group as compared to age-matched adults. Out of all pregnant women, 3 SARS-CoV-2-infected patients died, but all of them presented with comorbidities: asthma, hypertension, type 2 diabetes, autoimmune disease, and grade III obesity. In light of the increased rates of hospitalization and mortality, those authors concluded that pregnant women are more at risk for severe course of the disease as compared to their non-pregnant age peers [25]. 

Hematologic markers, which have been known to be elevated during COVID-19, include NLR (neutrophil-to-lymphocyte ratio) and PLR (platelet-to-lymphocyte ratio); and although LMR (lymphocyte-to-monocyte ratio) and LCR (lymphocyte-to-C-reactive-protein) have been assumed to be less alarming, they were demonstrated to be directly correlated with disease severity [26]. Blakeway et al. [27], found elevated levels of CRP and procalcitonin, together with lymphocytopenia and higher number of leukocytes, in laboratory findings of pregnant women [27]. A meta-analysis conducted by de Medeiros et al. [28], showed that the levels of lymphocytes decreased, while the levels of CRP increased [28]. Although the level of lymphocytes can vary during pregnancy, this can be indicative of a poor prognosis [3]. For this reason, CRP can be used as a biomarker of bacterial infection and may be associated with the risk of puerperal infection (data not evaluated) [29]. In our study, increased levels of leukocyte and neutrophils in controls as compared to the study group were surprising. Elevated neutrophil values are more often observed in the severe course of COVID–19 [30]. Possibly, it is the decreased level of neutrophils in the pregnant women or parturients which is responsible for a typically mild course of the disease, but the exact mechanism remains to be fully elucidated. Importantly, the SARS-CoV-2 infection modulates and disrupts protein synthesis in the body [31], which in some cases may affect neutrophil production. Importantly, elevated leukocyte and neutrophil count in the second half of pregnancy is not necessarily indicative of a pathology, as it may be a physiological reaction to the normal processes associated with gestation. Our results were consistent with the findings of Sun et al. [32], who demonstrated that blood indices of pregnant patients with COVID-19 showed a significantly lower lymphocyte count than in controls, i.e., pregnant women without COVID-19. Other blood parameters including neutrophil count and CRP levels, of the pregnant COVID-19 patients were significantly higher as compared to pregnant controls [32]. These results may be indicative of an inflammation and are typical clinical characteristics of pneumonia. 

Pregnancy is a known risk for venous thromboembolism characterized by a procoagulant imbalance [33]. In our study, we demonstrated elevated levels of D-dimers in pregnant women. COVID-19 may further enhance hypercoagulability in pregnant individuals, putting them at even greater risk for thromboembolism. Therefore, such test should be considered in pregnant women with confirmed SARS-CoV-2 infection [33]. 

It is not only the effect of COVID-19 on the maternal health, but also the fetal and neonatal condition which is the cause for concern. In our study, we demonstrated statistically significant differences in Apgar score at 1 and 5 minutes of life and cord blood ABG test. Neonates born to SARS-CoV-2-infected mothers presented with worse Apgar scores at 1 and 5 minutes of life and lower pH. The Apgar score is not designed to predict neonatal outcome outside the peripartum period. Regardless, low Apgar scores are associated with problems during the prenatal and peripartum period. The literature offers evidence of a relationship between low scores (<7) and subsequent respiratory distress, neurologic disability, and poor cognitive function later in life [34,35]. Hekimoğlu et al. [36], demonstrated an increase in the number of cases who required postpartum resuscitation, had a 5 min Apgar score of <7, and were treated with hypothermia due to a diagnosis of hypoxic-ischemic encephalopathy [36]. Neonatal Apgar score of ≥7 at 5 minutes of life may not be sufficient to verify the wellbeing of a newborn. Relying only on the Apgar scores may create the risk of not identifying newborns with mild metabolic acidosis. The need of routine ABG of the cord blood should be considered in prospective studies, even in the absence of fetal distress signs and with the Apgar score of ≥7. In our study, ABG test (pH) scores were lower in neonates born to SARS-CoV-2-infected mothers. 

The mode of delivery does not seem to affect the incidence of neonatal SARS-CoV-2 infections. In our study, we found no cases of SARS-CoV-2 infection in any of the neonates immediately upon birth, which is consistent with the findings of Gale et al. [21], who analyzed 116 infected mothers (44%—cesarean section and 56%—vaginal delivery) and found no cases of SARS-CoV-2 infection in their neonates over the course of 14 days [37]. 

Considering the available evidence, the healthcare personnel and the expectant mothers should be aware of the dangers and the possibility of non-specific symptoms and non-typical course of the SARS-CoV-2 infection. Therefore, it is imperative to promote preventive measures to lower the risk for transmission or severe course of the disease and the associated complications. In the event of an active infection, it is vital to monitor maternal and fetal wellbeing at high referral center and assemble an interdisciplinary team to implement all necessary measures. Importantly, one should bear in mind the speedy mutation of the virus and that the fact that the new variants might result in different symptoms and health problems.

Our conclusions allow to formulate some recommendations about the management of pregnant women with SARS–CoV-2. In light of the fact that we found no risk for vertical mother-to-child transmission of the SARS-CoV-2 virus, it is advisable to attempt vaginal delivery in cases with mild course of the disease and no pregnancy-related pathologies, as it decreases the risk for complications associated with elective cesarean sections. APTT, CRP and procalcitonin are the blood parameters which should be used to determine the severity of COVID–19 course in pregnant women. Maternal procalcitonin levels before delivery should be assayed to serve as predictive markers for neonatal condition in the first minutes of life.

### Strengths and Weaknesses of the Study

The fact that we included all SARS-CoV-2-infected women (who met the study inclusion and exclusion criteria) hospitalized at the time of the study is a definite strength. The exclusion criteria in the analysis were used to obtain reliable information on the relationship between SARS-CoV-2 infection and maternal as well as neonatal parameters. Processing data from only one hospital is another strength of our study as it lowered to the risk of bias associated with differences in data collection and hospital practices. Additionally, we excluded incomplete data. On the other hand, lack of data on maternal and neonatal condition in the subsequent days after the delivery is a definite limitation of our study. Furthermore, the reported data are intuitively limited to a short-term follow-up period. The use of basic statistical tests for data analysis is yet another limitation. Multivariate regression analysis would have improved the practical aspect of the study. 

## 5. Conclusions

Elective cesarean section is the most common mode of delivery for SARS-CoV2-infected mothers. Emergency cesarean sections are performed at earlier gestational age as compared to vaginal delivery and elective cesarean section.

Delivery during an active COVID-19 infection may negatively affect neonatal wellbeing in the first minutes after birth. Lower Apgar scores were observed in neonates born to SARS-CoV-2-infected mothers who required oxygen therapy and whose procalcitonin levels were elevated.

SARS-CoV-2 infection affects APTT and the levels of hemoglobin, erythrocyte, leukocyte, fibrinogen, and CRP. A relationship has been found between COVID-19 severity and APTT, as well as concentrations of CRP and procalcitonin. More severe course of the disease is associated with higher APTT, CRO and procalcitonin levels. The inflammatory markers and hematological parameters are reliable predictors for the severity of the disease and the prognosis, and they should be taken into consideration while providing care to SARS-CoV-2-infected expectant mothers. 

## Figures and Tables

**Table 1:** Characteristics of the study populations.

Parameters	Study Group (*N* = 304)		Control Group (*N* = 329)		*p*-Value
	M ± SD	Me (Min–Max)	M ± SD	Me (Min–Max)	
Age (years)	30.9 ± 5.0	31 (18–45)	29.9 ± 5.1	30 (17–44)	0.016
BMI (kg/m^2^)	28.8 ± 5.2	28 (18.3–48)	29.2 ± 4.4	28 (19–45.2)	0.170
Weight (kg)	80.7 ± 16.5	79 (50–139)	80.7 ± 13.0	75 (53–120)	0.832
Gestational week at hospital admission (weeks)	35.5 ± 7.1	38 (23–41)	39.0 ± 1.0	39 (33–42)	<0.0001
Comorbidities—*n* (%)	(*N* = 304)		(*N* = 329)		
Gestational diabetes grade G1	38 (12.50)		30 (9.11)		0.019
Gestational diabetes grade G2	6 (1.97)		7 (2.13)		
Hypertension	8 (2.63)		20 (6.08)		
Hypothyroidism	48 (15.79)		29 (8.81)		
Course of COVID-19—*n* (%)	(*N* = 304)		---		---
Mild	269 (88.49)		---		
Moderate	17 (5.59)		---		
Severe	18 (5.92)		---		
Oxygen therapy—*n* (%)	(*N* = 304)				---
No	273 (89.80)		---		
Yes	31 (10.20)		---		
Mode of delivery—*n* (%)	(*N* = 242)		(*N* = 329)		0.011
Spontaneous vaginal delivery (SVD)	44 (18.18)		257 (78.12)		
Elective cesarean section (ECS)	188 (77.69)		30 (9.12)		
Emergency cesarean section (EMCS)	6 (2.48)		29 (8.81)		
Vacuum extraction (VACCUM)	4 (1.65)		13 (3.95)		
Death during the perinatal period—*n* (%)	(*N* = 242)		(*N* = 329)		
No	240 (99.17)		329 (100.00)		0.098
Yes	2 (0.83)		0 (0.00)		

M—mean; SD—standard deviation; Me—median; BMI—body mass index.

**Table 2:** Maternal blood parameters.

Parameter	Study Group (*N* = 304)		Control Group (*N* = 329)		*p*-Value
	M ± SD	Me (Min–Max)	M ± SD	Me (Min–Max)	
Hemoglobin (mmol/L)	7.5 ± 0.8	7.6 (5.4–10.5)	6.7 ± 0.9	6.8 (3.5–8.8)	0.000
Erythrocyte(T/L)	4.1 ± 0.4	4.1 (2.8–5.8)	3.7 ± 1.9	3.6 (1.7–4.8)	0.000
Leukocyte (G/L)	9.7 ± 4.0	9.1 (3.6–34.6)	13.3 ± 3.9	12.9 (4.4–42.2)	0.000
Neutrophil (%)	74.1 ± 7.6	74.2 (46.9–92.6)	75.2 ± 7.6	75.9 (10.1–96.3)	0.025
Fibrinogen (G/L)	4.3 ± 1.0	4.2 (0.6–6.7)	3.7 ± 0.8	3.7 (0.5–6.8)	0.000
APTT (seconds)	29.1 ± 3.4	28.6 (22.6–44.5)	30.8 ± 3.4	30.1 (20.8–53.4)	0.000
CRP (mg/L)	22.1 ± 32.5	7.6 (0.6–194.5)	9.8 ± 21.6	4.0 (0.4–142.3)	0.000
Procalcitonin (ng/mL)	1.2 ± 3.7	0.1 (0.02–20.2)	0.3 ± 0.5	0.1 (0.002–1.4)	0.988
D-dimer (µg/L)	3244 ± 5856	1655 (418–41,922)	---	---	---

M—mean; SD—standard deviation; Me—median; APTT—activated partial thromboplastin; CRP—C-Reactive Protein.

**Table 3:** Severity of COVID-19 in pregnant women (*N* = 304) versus selected maternal blood parameters.

Parameter	Mild Course (*N* = 269)		Moderate Course (*N* = 17)		Severe Course (*N* = 18)		*p*-Value
	M ±SD	Me (Min–Max)	M ± SD	Me (Min–Max)	M ± SD	Me (Min–Max)	
APTT	28.18 ± 2.7	28 (22.6–36.4)	32 ± 4.2	31 (26.1–39.9)	32.8 ± 10	27.6 (26.3–44.5)	0.020
CRP	16.0 ± 27.4	5.4 (0.6–194.5)	50.2 ±30.0	45.5 (17.1–110.1)	66.78 ± 42.1	66.78 (0.6–136)	0.000
Procalcitonin	0.07 ± 0.08	0.03 (0.02–0.32)	0.47 ± 0.4	0.22 (0.07–1.01)	1.5 ± 1.8	1.5 (0.24–3.65)	0.032

M—mean; SD—standard deviation; Me—median; APTT—activated partial thromboplastin; CRP—C-Reactive Protein.

**Table 4:** Selected neonatal parameters in the study population.

Parameters	Study Group (*N* = 242)		Control Group (*N* = 329)		*p*-Value
	M ± SD	Me (Min–Max)	M ± SD	Me (Min–Max)	
Apgar 1′	9.3 ± 1.73	10 (0–10)	9.8 ± 0.67	10 (5–10)	0.000
Apgar 5′	9.5 ± 1.74	10 (0–10)	9.9 ± 0.36	10 (7–10)	0.000
ABG test (pH)	7.3 ± 0.06	7.320 (6.68–7.45)	7.32 ± 0.23	7.350 (3.370–7.500)	0.016

M—mean; SD—standard deviation; Me—median; ABG—Arterial Blood Gas.

**Table 5:** Selected blood parameters of SARS-CoV-2-infected pregnant women versus neonatal condition assessed with Apgar score.

Pairs of Variables (*N* = 242)	R Spearman	t(*N* − 2)	*p*-Value
D-dimer & Apgar 1′	0.234294	1.80349	0.076
D-dimer & Apgar 5′	0.174482	1.31415	0.194
Fibrinogen & Apgar 1′	0.171710	1.30434	0.197
Fibrinogen & Apgar 5′	0.101214	0.75449	0.453
APTT & Apgar 1′	−0.214552	−1.64384	0.105
APTT & Apgar 5′	−0.229382	−1.74774	0.086
CRP & Apgar 1′	−0.129546	−1.64739	0.101
CRP & Apgar 5′	−0.086987	−1.07653	0.283
Procalcitonin & Apgar 1′	−0.444997	−2.16597	0.043
Procalcitonin & Apgar 5′	−0.344115	−1.59753	0.126

APTT—activated partial thromboplastin; CRP—C-Reactive Protein.

## Data Availability

The data presented in this study are available on request from the corresponding author.

## Abbreviations

ABG	Arterial Blood Gas
APTT	activated partial thromboplastin
ARDS	acute respiratory distress syndrome
BMI	body mass index
COVID-19	Corona Virus Disease 2019
CRP	C-Reactive Protein
ECS	elective cesarean section
EMCS	emergency cesarean section
RT-PCR	reverse transcription polymerase chain reaction
SARS-CoV-2	severe acute respiratory syndrome coronavirus 2
SpO_2_	oxygen saturation
SVD	spontaneous vaginal delivery
VACCUM	vacuum extraction
